# Supplementary material for: FOSB–PCDHB13 Axis Disrupts the Microtubule Network in Non-Small Cell Lung Cancer
Source: Cancers (Basel). 2019 Jan 17;11(1):107. doi: 10.3390/cancers11010107 (PMC6357195; doi:10.3390/cancers11010107)

Supplementary Materials: FOSB–PCDHB13 Axis Disrupts the Microtubule Network in Non-Small Cell Lung Cancer

Chen-Hung Ting, Kang-Yun Lee, Sheng-Ming Wu, Po-Hao Feng, Yao-Fei Chan, Yi-Chun Chen and Jyh-Yih Chen


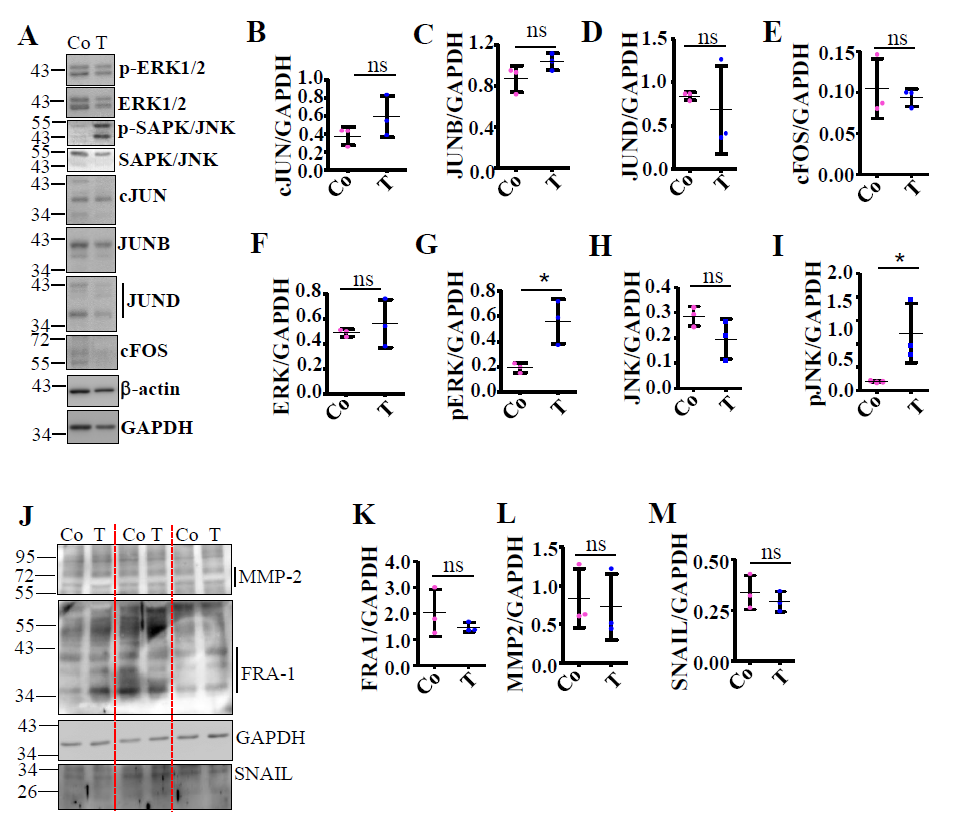


**Figure S1:** Protein expressions of AP-1 members in A549 cells. (**A**,**J**) Total lysates from A549 cells without (Co) or with TP4 treatment (T) were analyzed by Western blot using antibodies against GAPDH, AP-1 members (c-JUN, JUNB, JUND, c-FOS, FRA1), MAP kinases (ERK1/2, phospho-ERK1/2, SAPK/JNK, phospho-SAPK/JNK), SNAIL, and MMP2. (**B−I, K−M**) Quantitative analysis of the blots is shown in (**A**,**J**) normalized to GAPDH. Results represent the mean ± SD (*n* = 3, two-tailed *t*-test: * *p* < 0.05 versus Control).


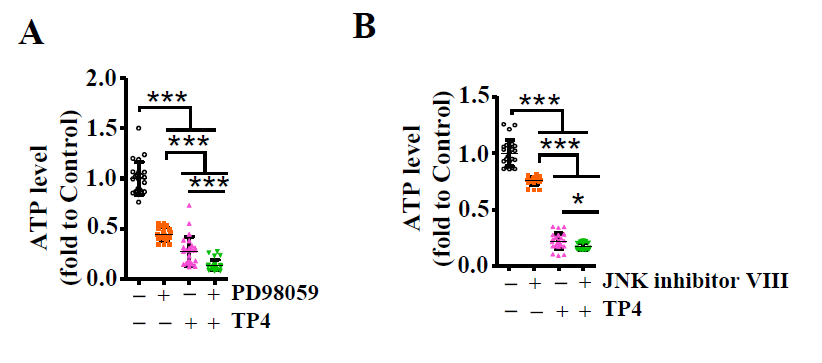


**Figure S2:** Inhibition of MAPK signaling enhanced TP4 cytotoxicity in A549 cells. (**A,B**) Cell viability was measured in cells treated with PD98059 (**A**) or JNK inhibitor VIII (**B**) and TP4. Eight wells were analyzed for each assay. Results represent the mean ± SD. (*n* = 3, two-tailed *t*-test: * *p* < 0.05; *** *p* < 0.001).


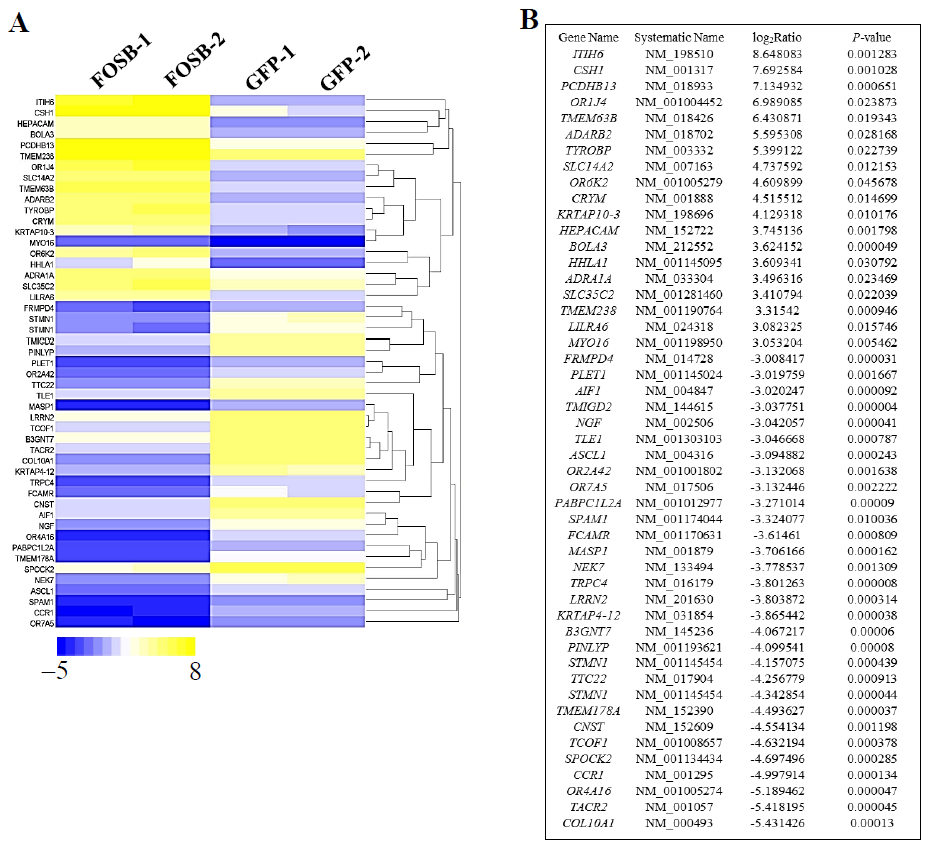


**Figure S3:** Gene expression profiling in FOSB-overexpressing A549 cells. (**A**) Heat map depicting expression changes of genes in GFP- or FOSB-tGFP-transfected A549 cells (scale bar indicates log_2_-fold changes). GFP-1, GFP-2 and FOSB-1, FOSB-2 indicate duplicate samples collected from two independent assays. (**B**) List of the differentially expressed genes (with log_2_ ratio > 3 or < −3) in GFP- or FOSB-transfected A549 cells. The P values of each identified gene are indicated.


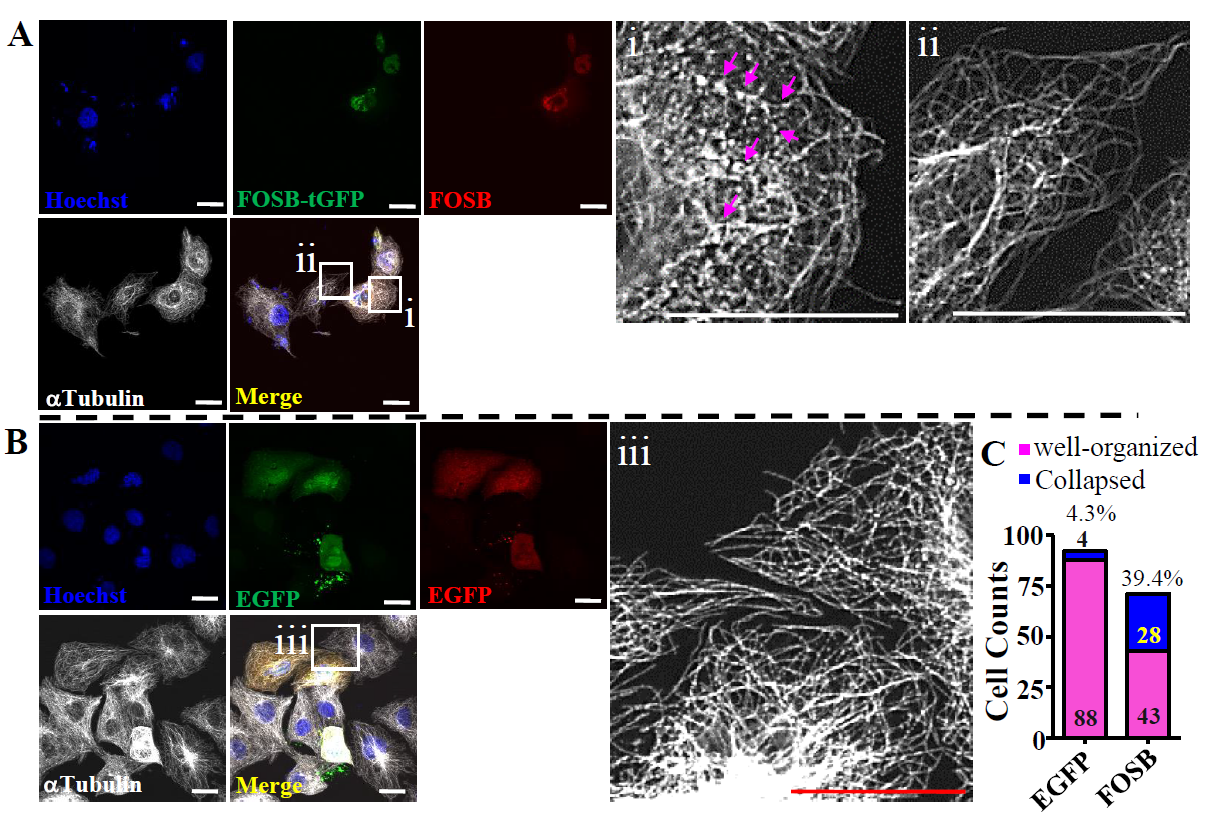


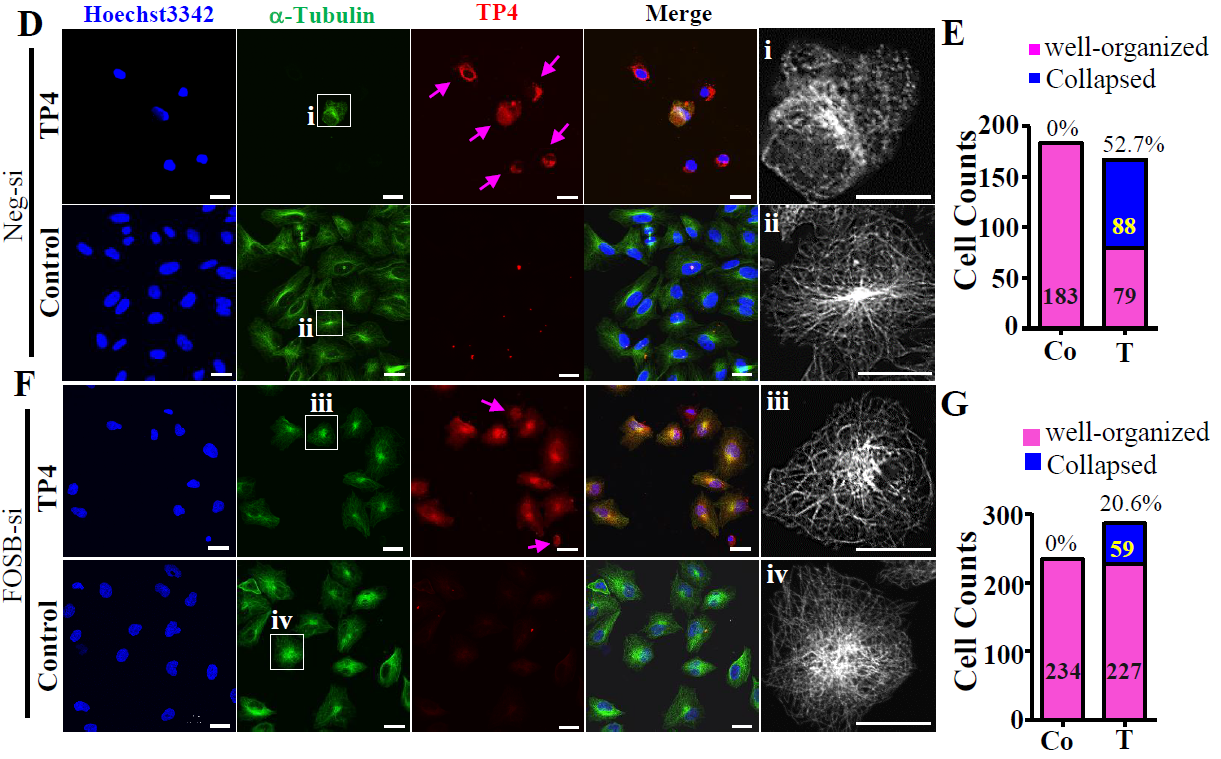


**Figure S4:** The microtubule cytoskeleton was affected by FOSB activation and TP4 treatment. (**A,B**) FOSB, EGFP, and α-Tubulin antibody staining is shown in red (FOSB/EGFP) or white (α-Tubulin) in A549 cells. Hoechst33342 was used for nuclei staining. Boxed regions (i and ii in **A;** and iii in **B**) are magnified in the panels to the right of the merged images. Pink arrows shown in box Ai indicate abnormal microtubule network. Bar: 20 μm. (**C**) Cell counts from the FOSB (**A,** *n* = 92 cells collected from two independent transfectants) or EGFP (**B**, *n* = 71 cells collected from two independent transfectants) transfected cells with well-organized or collapsed microtubule network. (**D, F**) TP4 and α-Tubulin antibody staining is shown in red (TP4) or green/white (α-Tubulin) in A549 cells transfected with control siRNAs (D) or FOSB siRNAs (**F**) received control (Co) or TP4 treatment (6.71 μM) for 3 h. Hoechst33342 was used for nuclei staining. Boxed regions are magnified in the panels to the right (i and ii in **D**, iii and iv in **F**). Arrows indicated cells defect in microtubule network. Bar: 20 μm. (**E,G**) Cell counts from the control siRNA-transfected cells receiving control/TP4 treatments (**E**, *n* = 183 collected from control (Co)-treated cells and *n* = 167 collected from TP4-treated cells from three independent treatments) and from the FOSB siRNA-transfected cells receiving control (Co) /TP4 (T)-treated groups (**G**, *n* = 234 collected from control-treated cells and *n* = 286 collected from TP4-treated cells from three independent treatments) with well-organized or collapsed microtubule network.


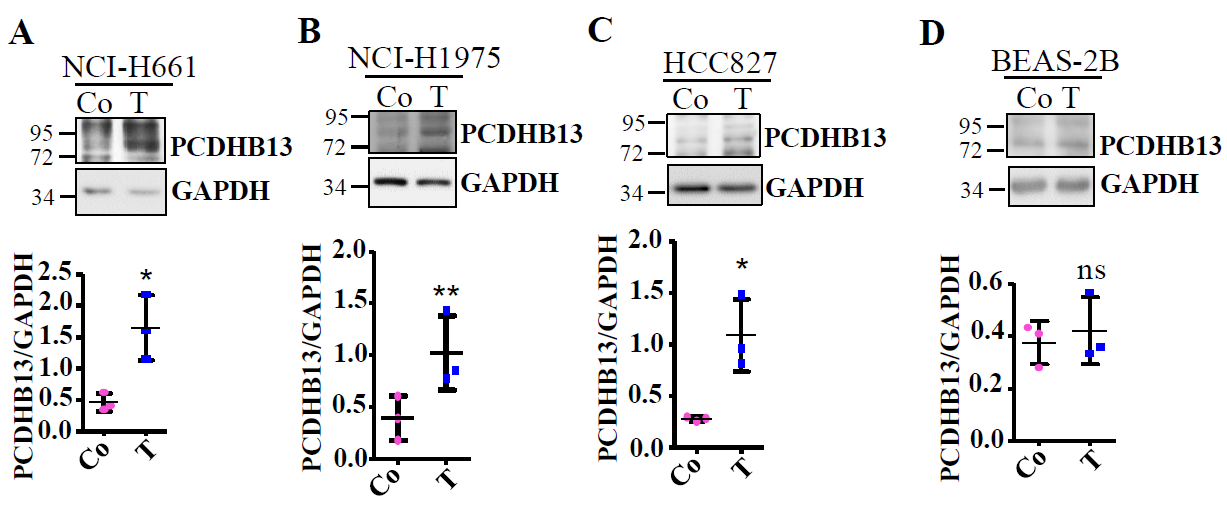


**Figure 5.** PCDHB13 was induced by TP4 in NSCLC cells. (**A−D**) Total lysates from NCI-H661 (**A**), NCI-H1975 (**B**), HCC827 (**C**) and BEAS-2B (**D**) cells without (Co) or with TP4 treatment (T) were analyzed by Western blot using antibodies against GAPDH and PCDHB13. Blots were quantified and normalized to GAPDH. Results represent the mean ± SD. (*n* = 3, two-tailed *t*-test: * *p* < 0.05; ** *p* < 0.01).


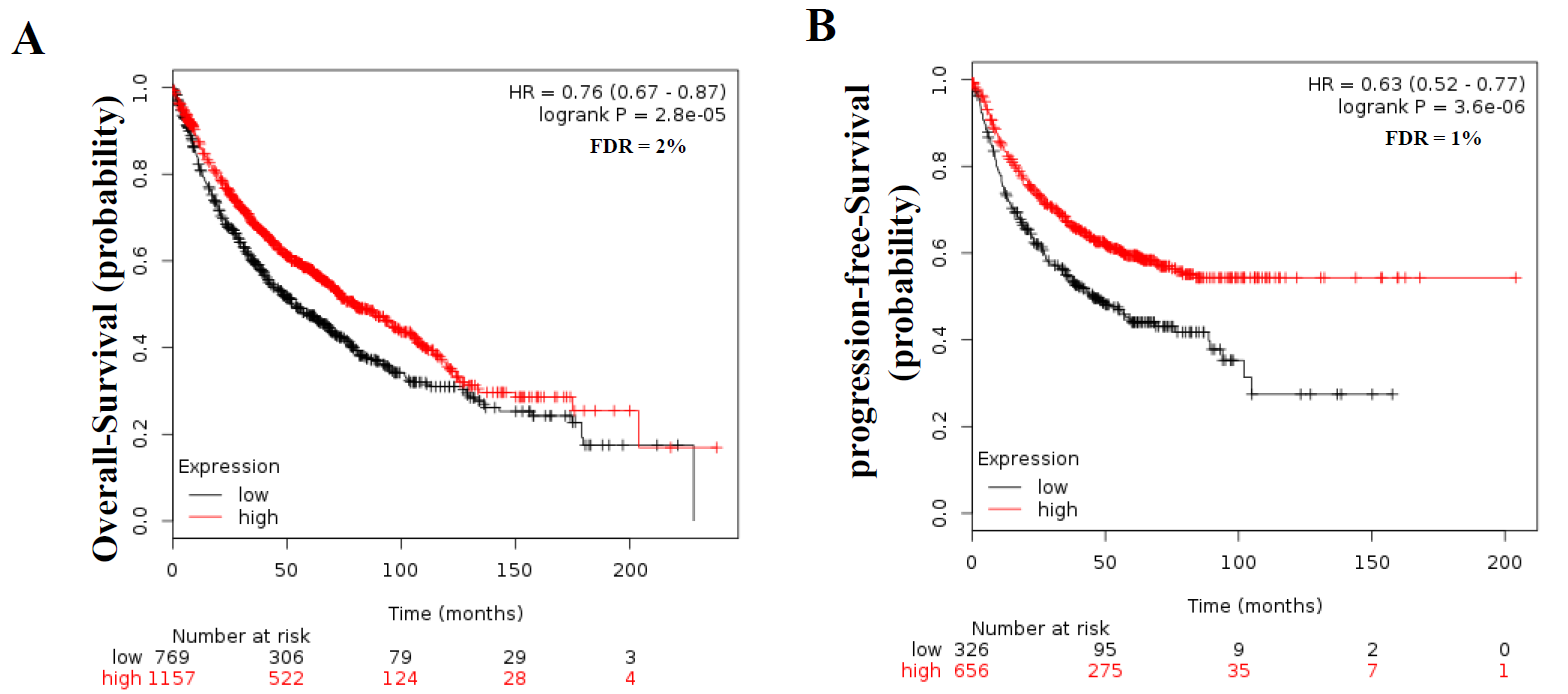


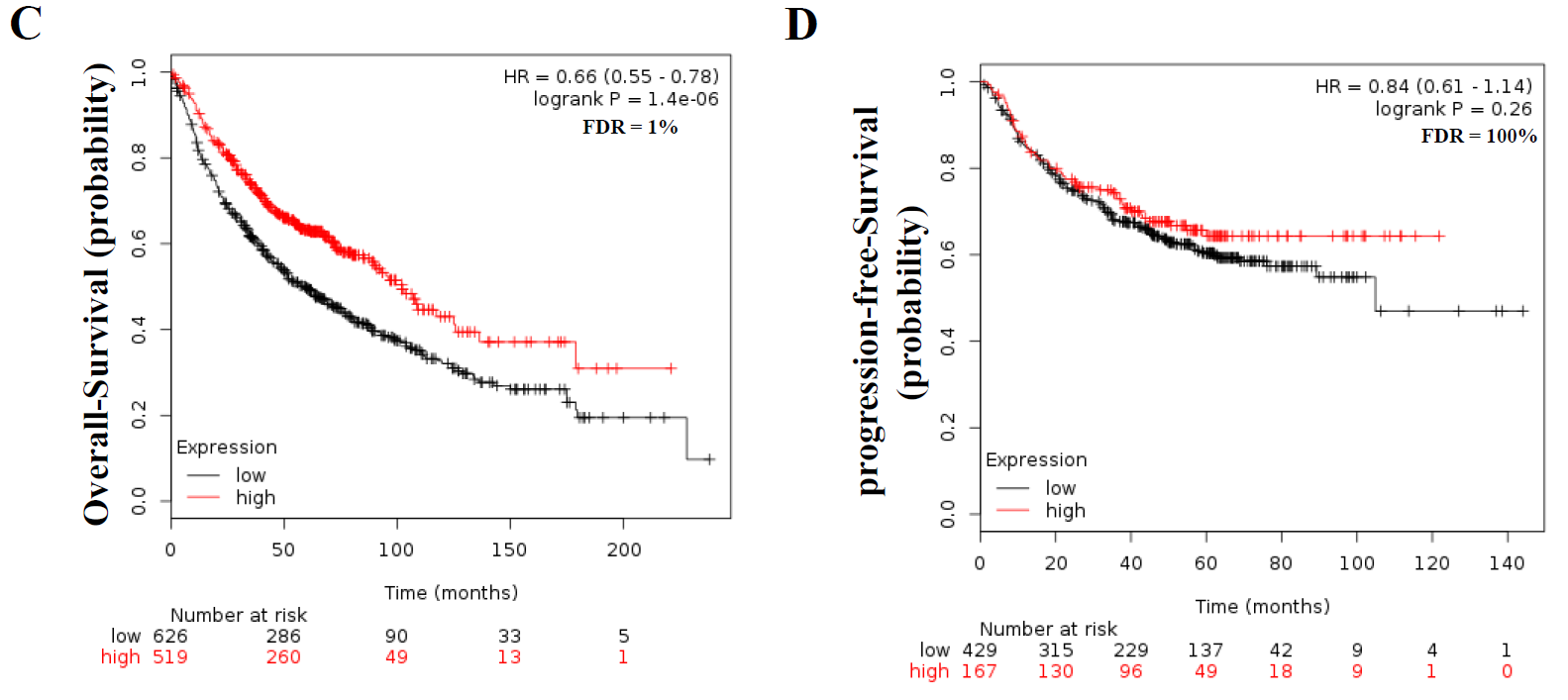


**Figure S6:** High expression of FOSB and PCDHB13 are associated with enhanced survival status in lung cancer patients. (**A−D**) Kaplan-Meier analysis of overall survival (OS) and progression-free survival (FPS) of lung cancer patients (*n* = 1928 and *n* = 982, respectively) correlation to FOSB (**A**,**B**) and PCDHB13 (**C**,**D**) high and low expression subgroups through Kaplan-Meier plotter analyses (<http://kmplot.com>). The data was processed using the ″auto-select best cutoff″ option in the analysis software to compute all possible cutoff values between the lower and upper quartiles. The FDR (false-discovery-rate) value are shown as indicated. *p* = 0. 000028 in (**A**); *p* = 0.0000036 in (**B**); *p* = 0.0000014 in (**C**); *p* = 0.26 in (**D**).


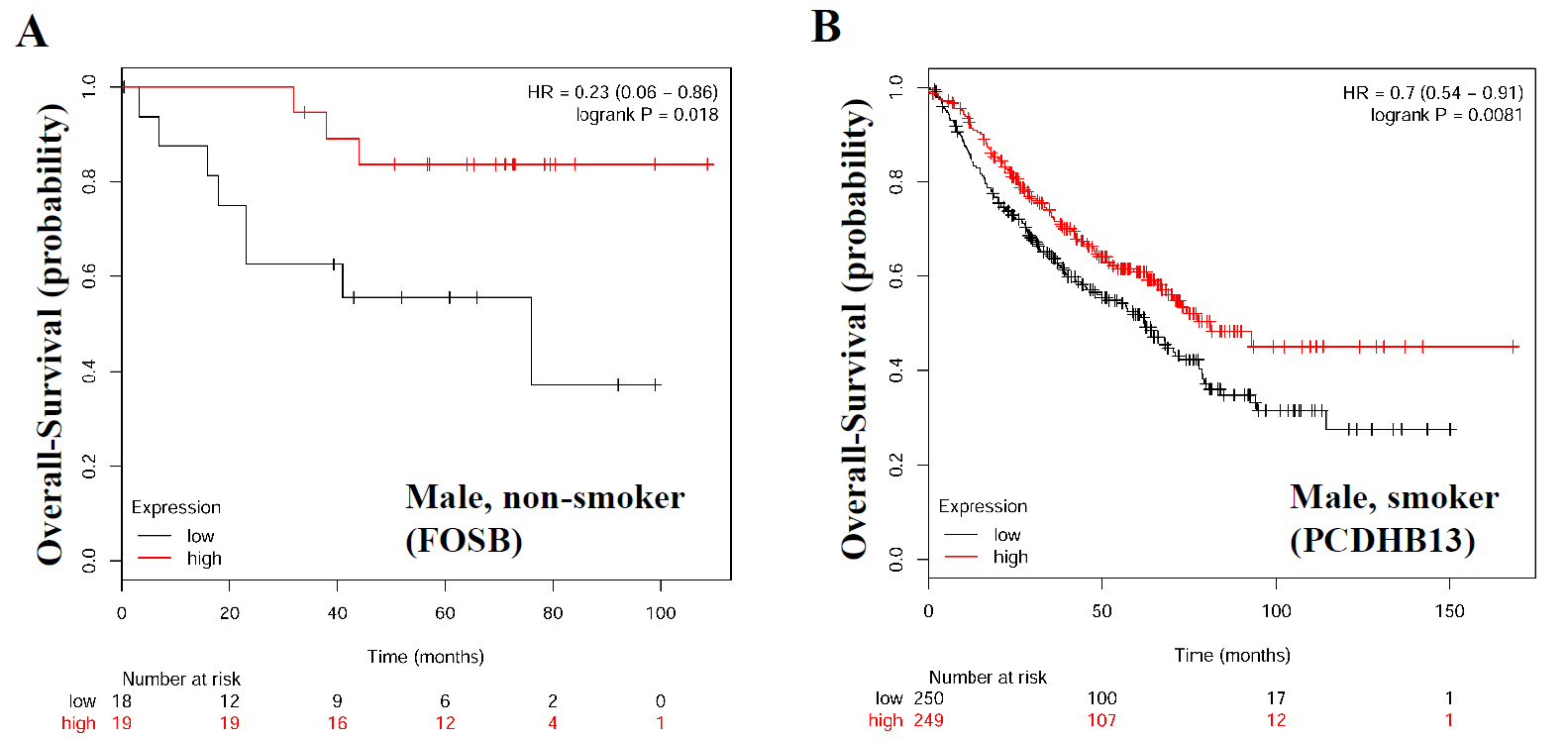


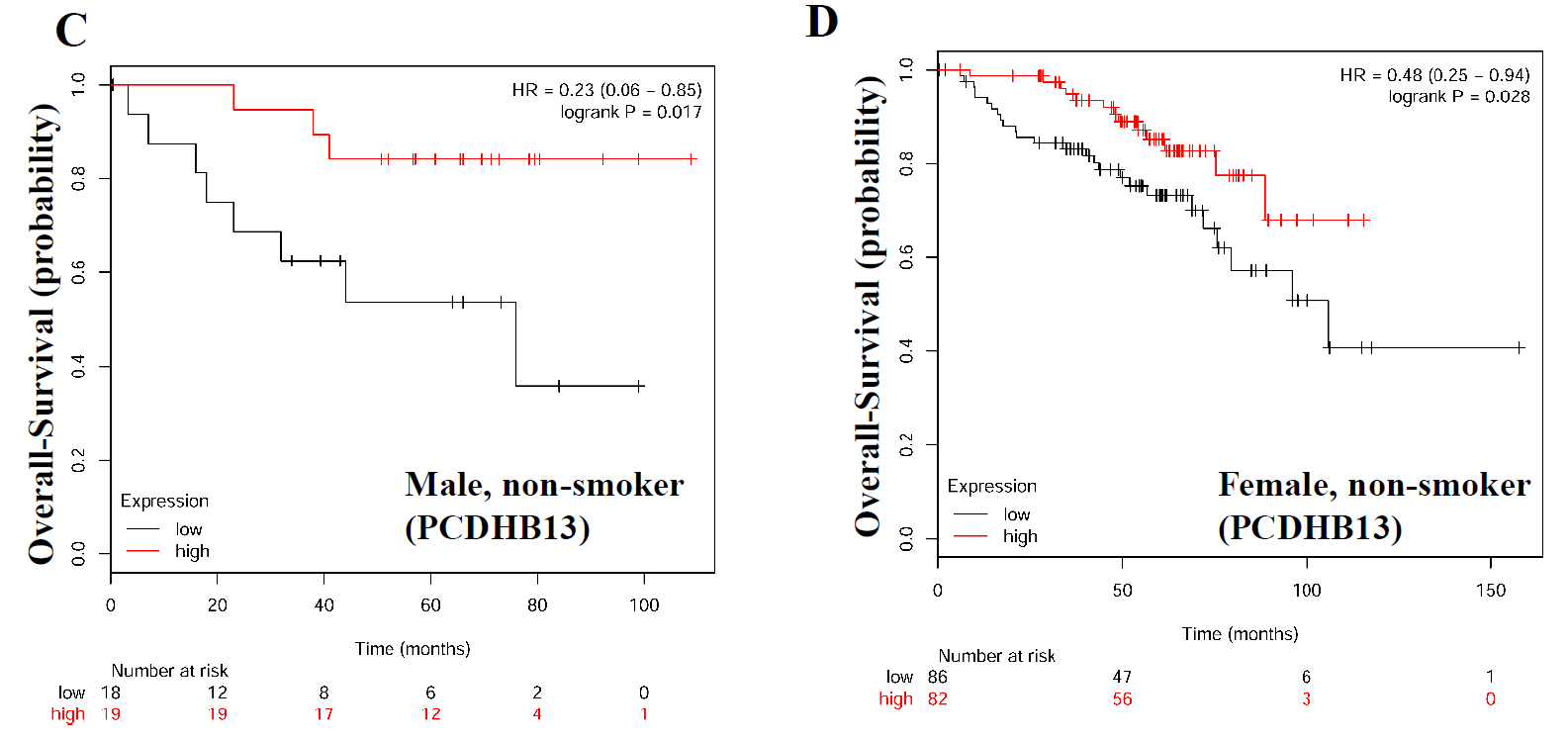


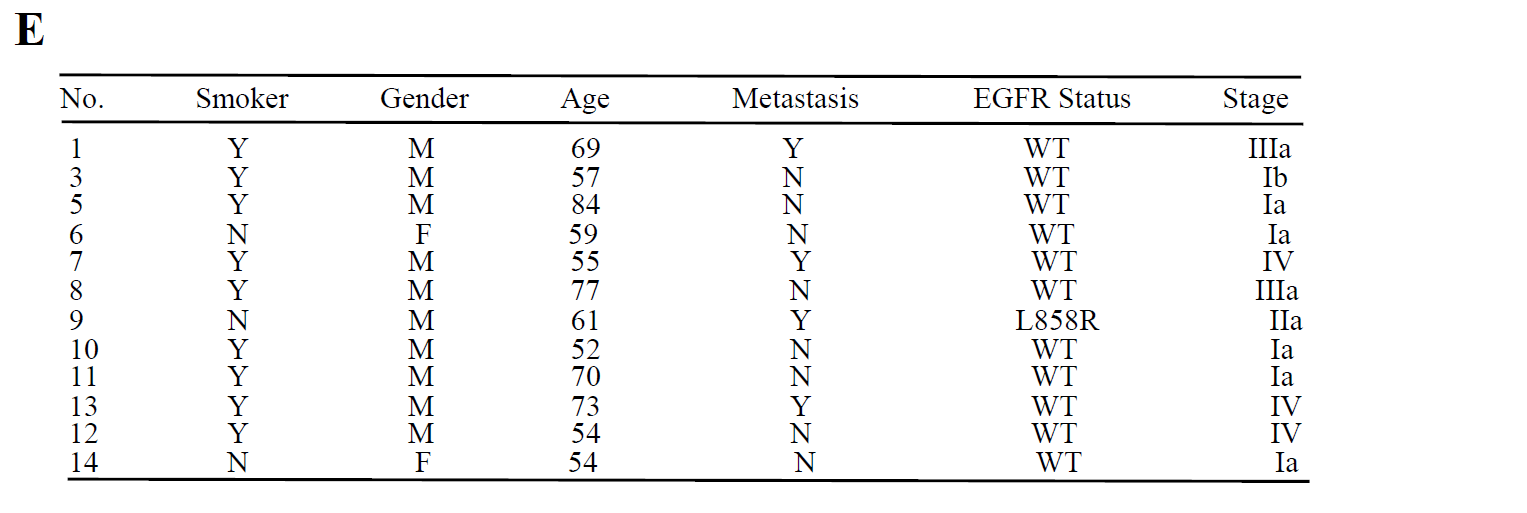


**Figure S7:** High expression of FOSB and PCDHB13 are associated with enhanced OS rate in different genders and smoking status of lung cancer patients. (**A−D**) Kaplan-Meier analysis of OS of lung cancer patients correlation to FOSB (**A**) and PCDHB13 (**B**−**D**) high and low expression subgroups through Kaplan-Meier plotter analyses (<http://kmplot.com>). *p* = 0. 018 in (**A**, male, non-smoker); *p* = 0.0081 in (**B**, male, smoker); *p* = 0.0017 in (C, male, non-smoker); *p* = 0.028, female, non-smoker in (**D**). (**E**) Characterizations of the disease status of twelve NSCLC patients in this study.


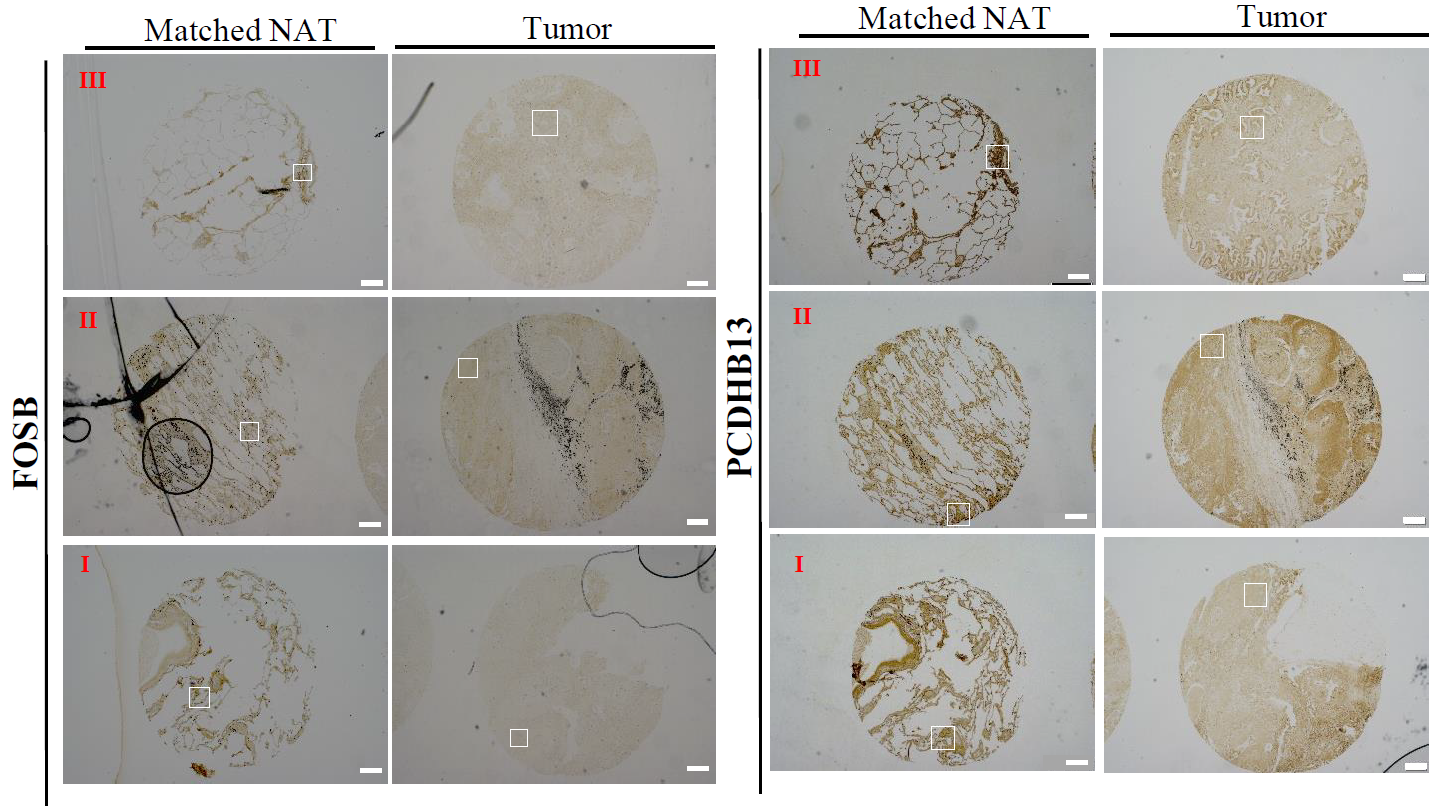


**Figure 8.** Complete uncropped tumor tissue images related to Figure 7A.


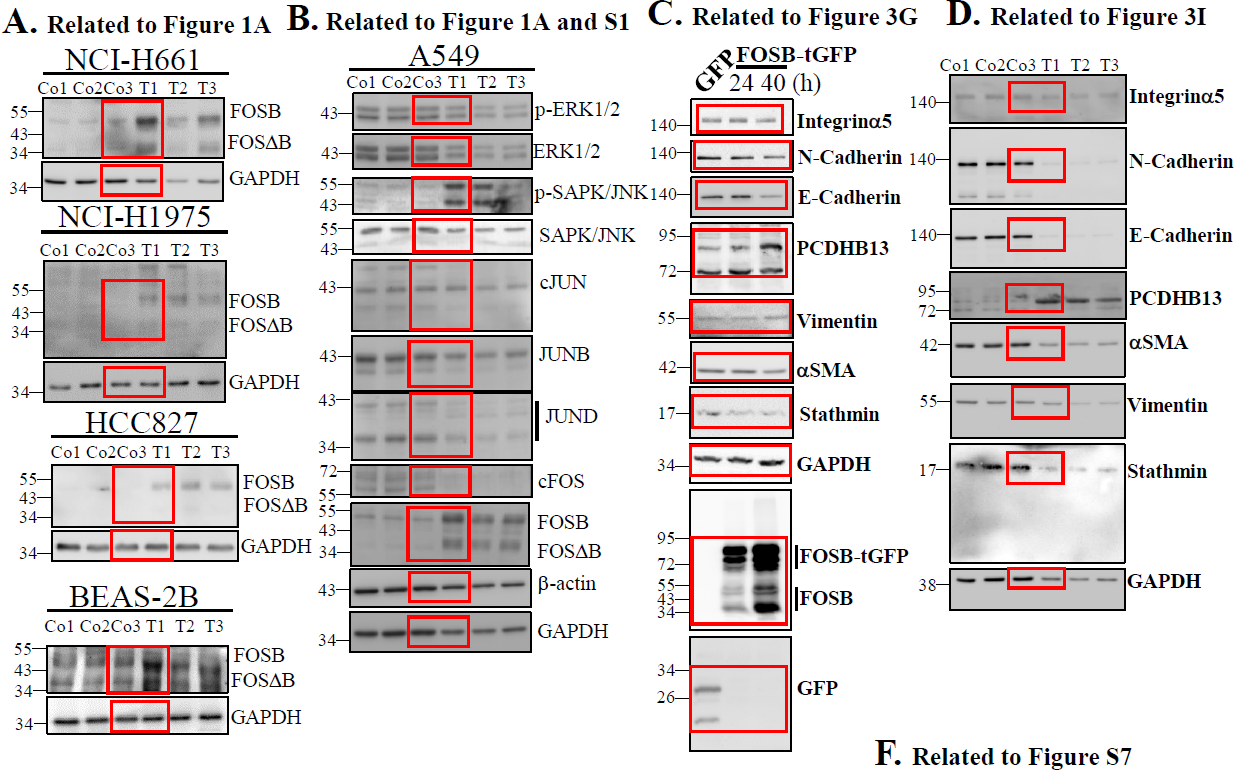


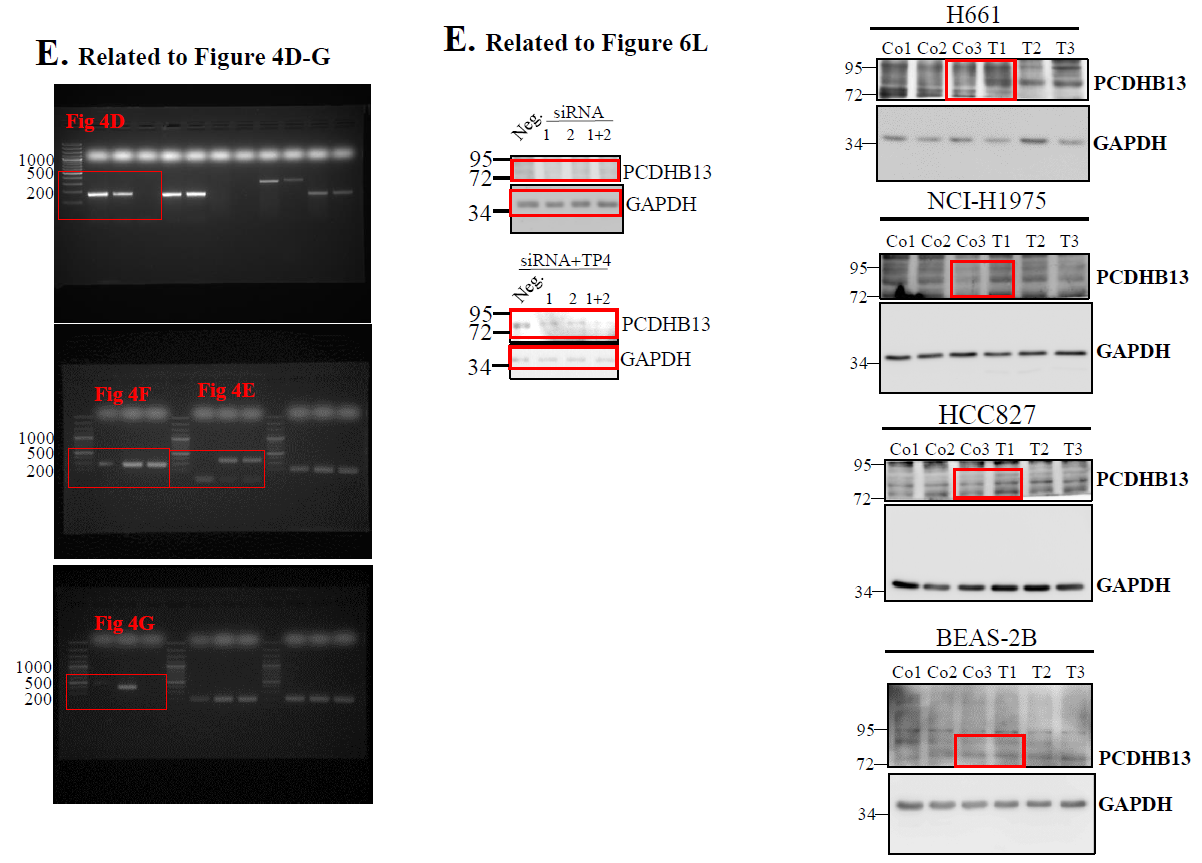


**Figure 9.** Complete and unedited Western blot images.

**Video S1:** Association of PCDHB13 with microtubules. Cytoskeletal projections of each protein were simulated by Imaris software.

© 2019 by the authors. Licensee MDPI, Basel, Switzerland. This article is an open access article distributed under the terms and conditions of the Creative Commons Attribution (CC BY) license (http://creativecommons.org/licenses/by/4.0/).
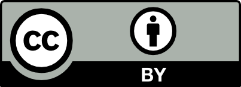

Supplement: Supplementary file 1 [file cancers-11-00107-s001.zip › suppl-layout/cancers-419604-suppl.docx]
